# Are pit latrines in urban areas of Sub-Saharan Africa performing? A review of usage, filling, insects and odour nuisances

**DOI:** 10.1186/s12889-016-2772-z

**Published:** 2016-02-04

**Authors:** Anne Nakagiri, Charles B. Niwagaba, Philip M. Nyenje, Robinah N. Kulabako, John B. Tumuhairwe, Frank Kansiime

**Affiliations:** 1 Department of Civil and Environmental Engineering, Makerere University, P.O Box 7062, Kampala, Uganda; 2 Department of Agricultural Production, Makerere University, P.O Box 7062, Kampala, Uganda; 3 Department of Environmental Management, Makerere University, P.O Box 7062, Kampala, Uganda

**Keywords:** Filling, Insects, Pit latrine, Smell, Sub- Saharan Africa usage

## Abstract

**Background:**

A pit latrine is the most basic form of improved sanitation which is currently used by a number of people around the globe. In spite of the wide spread use, known successes and advantages associated with pit latrines, they have received little attention in form of research and development. This review focuses on the usage and performance (filling, smell and insect nuisance) of pit latrines in urban areas of sub-Saharan Africa (SSA) and proposes approaches for their improvements and sustainability.

**Methods:**

Current pit latrine usage within urban SSA was calculated from Joint Monitoring Programme (JMP) water and sanitation country-files. We conducted a literature search and review of documents on pit latrine usage, filling, smell and insect nuisances in urban areas of SSA. Findings of the review are presented and discussed in this paper.

**Results and Discussion:**

Pit latrines are in use by more than half the urban population in SSA and especially among low income earners. An additional 36 million people in urban areas of SSA have adopted the pit latrine since 2007. However, their performance is unsatisfactory. Available literature shows that contributions have been made to address shortfalls related to pit latrine use in terms of science and technological innovations. However, further research is still needed.

**Conclusion:**

Any technology and process management innovations to pit latrines should involve scientifically guided approaches. In addition, development, dissemination and enforcement of minimum pit latrine design standards are important while the importance of hygienic latrines should also be emphasized.

**Electronic supplementary material:**

The online version of this article (doi:10.1186/s12889-016-2772-z) contains supplementary material, which is available to authorized users.

## Background

Globally, providing adequate sanitation is a challenge and the situation is worse in developing countries. Improved sanitation protects the environment and improves people’s health, thereby translating into socio-economic development and poverty eradication [1–3]. Access to improved sanitation worldwide stands at 64 %, with the lowest coverage of 41 % in urban areas of Sub-Saharan Africa (SSA) [4].

Sanitation provision in urban areas of SSA is predominantly on-site [5]. A number of technologies are currently in use, each of varying affordability, suitability, adaptability and user satisfaction. These technologies include septic tanks, aqua privies, biogas latrines, composting or dehydrating toilets and pit latrines. The use of septic tanks in SSA currently stands at only 5 % of the population [6]. Challenges with the use of septic tanks are mainly high construction costs, space limitations, lack of water and blockages that result from use of bulk materials for anal cleansing. The performance of aqua privies in SSA has been unsatisfactory. In Ghana, where the aqua privy was once widely used, it is now considered a failed technology at a national level. The uncontrolled odours [7], social/cultural issues and water shortages led to the abandonment of the aqua privy technology [8] in Ghana.

Biogas latrines have been installed as communal/public facilities in some areas of SSA [9, 10]. Their initial cost and operational skill requirements are beyond household level applications. Further, insufficient biogas to meet cooking requirements, gas leakage and the cultural issues with end-use of the slurry have hindered their adoption at household level. Replication or up-scaling composting or dehydrating toilets in SSA has registered varying levels of success. In east and southern Africa, cultural acceptance and misuse of the facilities have been cited as challenges to their use [11]. In Ghana, failure of the Enviroloo, a type of composting toilet was caused by lack of readily available spare parts for repairing fans that were located on top of their chimney pipes [7]. The success and failure attributes of the different sanitation technologies used in Sub-Saharan Africa are summarised in Additional file 1: Table S1.

Pit latrines still remain widely used and are the commonest basic form of improved sanitation [2]. Of the 2.7 billion people using on-site sanitation worldwide [6], an estimated 1.77 billion use some form of pit latrine as their primary means of excreta disposal [12]. Low-cost, simplicity of construction, little or no water usage, and ease in operation and maintenance, the ability to cope with bulky varied anal cleansing materials and the ease for regular improvement of the facility makes it convenient and easily taken up. The pit latrine technology currently offers a number of options ranging from simple designs like the traditional (without concrete slabs) to the simple improved, and further to more advanced Ventilated Improved (VIP), Reed Odourless Earth Closet (ROEC), pour flush and borehole pit latrines. However, the use of pit latrines in urban areas of SSA has been marred by poor performance in terms of fast filling, bad smells and insect nuisances, which are associated with user dissatisfaction and a risk to disease transmission. Yet, well-constructed, operated and maintained pit latrines isolate, store and partially treat human excreta thereby minimising contact and their inherent public health hazards. In spite of the known successes and advantages associated with pit latrines, they have received little attention inform of research and development. The wide spread application and use of pit latrines necessitates sufficient knowledge of their performance in order to develop, design and operate them better, thereby improving the sanitation situation of the users. This paper reviews previous and current knowledge on pit latrines usage and performance in urban areas of SSA. Knowledge gaps are identified and strategies or interventions that may improve the performance and sustainability of pit latrines are suggested. The performance elements covered in this review are pit latrine filling, smell and insect nuisances.

## Methods

**Fig. 1:**
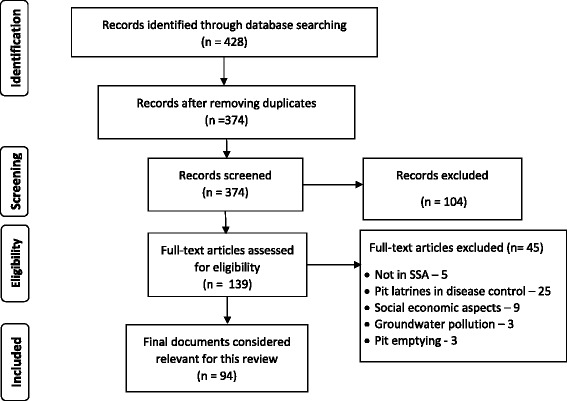
PRISMA flow diagram of the review inclusion and exclusion process

We undertook a comprehensive literature search according to PRISMAS guidelines [13], as shown in Fig. 1, to find relevant documents both published and unpublished, with no date restriction (Fig. 1). This was because the review covers past and present knowledge, on pit latrines. We searched Google (http://www.google.com/), Google scholar (https://scholar.google.com/) and Science Direct (http://www.sciencedirect.com/) using the following keywords: “pit latrine”, “pit privy”, “pit latrine performance”, “Pit latrine + sanitation”, “Pit latrine filling smell and insects”, “pit latrine filling + sub-Saharan Africa” “pit latrine smell + sub-Saharan Africa”, “pit latrine + mosquitoes”, “Pit latrine flies + sub-Saharan Africa”, “Sanitation policy + sub-Saharan Africa”. The titles of retrieved articles were read to exclude duplication ahead of the screening process. During screening, the titles and abstracts of the documents were read to determine their eligibility of articles for full text assessment. In case of sanitation articles and reports, the complete document was obtained and scanned through to determine its eligibility. Documents selected for full text assessment were those that had information pour/flush to pit; ventilated improved pit latrines; pit latrines with concrete slabs; traditional latrines (pit latrines with slabs not made of concrete); pit latrines without slabs/open pits (as unimproved latrines). At full text assessment, the contents of the document were critically examined to identify information on the history of pit latrines (no restriction of location), their usage, topics that covered smell and insect nuisances (limited to SSA) and those were then considered relevant for the review. In addition, references in articles and reports guided further inquiry and review. Information from the selected articles was extracted and the findings were used to develop this review. A figure on pit latrine and sanitation development milestones was developed from dates sited in literature. The pit latrine usage in different countries across SSA was determined based on available WHO/UNICEF survey data on estimates on the use of sanitation facilities for the different countries of SSA [14] and the figures were then used to develop the map on pit latrines usage. The data source used for each country is indicated in Additional file 1: Table S2.

## Results and Discussion

### History of the pit latrine technology

The practice of human excreta disposal in the ground is a simple sanitation solution that has been used for thousands of years. Burying excreta in shallow holes referred to as the cat method and crude forms of pit latrines where horizontal logs were placed across the holes for support during use have been reported [15–17]. These human excreta disposal solutions did not require any technical construction. Although these technologies are still used in some developing countries, and are better human excreta disposal systems than open defecation, they are unimproved. The danger of contact with the excreta by humans, animals, and vectors of disease transmission plus soil contamination remain high.

**Fig. 2:**
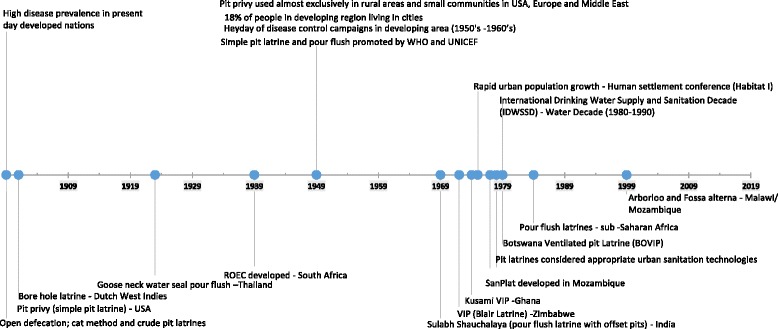
Pit latrine and sanitation development milestones

The historical use of technical pit latrine designs dates to the early 20th century. They were developed and promoted in rural and small communities of present day developed nations to minimise indiscriminate pollution of the environment with human excreta that had resulted in high incidences of diseases. One very important World Health Organisation publication by EG Wagner and JN Lanoix [18] in the late 1905’s details technical data on pit latrines and ways of achieving successful human excreta disposal programs. The basic components of the pit latrine design are a hole dug in the ground in which excreta and anal cleansing material is deposited, a slab with a drop hole that covers the pit and a superstructure for privacy [19, 20]. To date, a number of design incorporations and modifications to the pit latrine have been developed, (Fig. 2) each targeted to performance improvement, and the socio-economic status of the communities.

One such design, the borehole latrine design with small cross-sectional pit diameter (300–500 mm) evolved during the early 20th century in the Dutch East Indies. The basis of this pit latrine design is not documented. However, it was noted that borehole latrines were at times included in kits prepared for disasters as they can be quickly and easily dug [17]. In order to mitigate the odour and insects, a water seal by the goose neck pour flush was developed in Thailand in the 1920’s. Another advanced pit latrine design aimed at addressing odour and insect problems of simple pit latrines is the Reed Odourless Earth Closet (ROEC) developed in South Africa in 1940’s [21].

The use of a simple pit latrine in SSA dates to the 1950’s–1960’s, during the heyday of the disease control campaigns. However, the pit latrine was mainly promoted for use in rural areas [18, 22, 23]. The major health and aesthetic problems associated with pit latrines then were insects (flies and mosquitoes) and odours [21]. To overcome these shortfalls, the ventilated improved pit latrine (VIP), initially called the Blair Latrine, was developed in Zimbabwe in the early 1970’s. Modifications to the VIP made to date include the Kusami Ventilated improved pit (KVIP) in Ghana [24, 25] and the ‘Revised Earth Closet II’ (REC II), also known as the Ventilated Improved Double Pit (VIDP) latrine in Botswana [26, 27]. In an effort to mitigate insect, odour and cost challenges of VIP latrines, another innovation design, the SanPlat was developed in Mozambique in 1979 [28].

Towards the late 1970’s, sanitation and health crises in developing nations were a result of rapid urban population growth and ‘exploding cities’. For instance, up to 70 % of new inhabitants in some African cities were residing in slums and shantytowns without amenities [29]. The World Bank thus undertook research with emphasis directed towards low cost sanitation alternatives to sewerage. The results of the research, presented in a series of publications consider pit latrines as appropriate technologies for waste disposal in developing countries [30–32]. Some pit latrine designs were then recommended as appropriate sanitation technologies for urban areas. Pit latrine were thereafter, accepted, adopted, promoted and used in urban areas of different countries in SSA during the Water Decade [20, 29]. Currently, in the 21st century, interest in pit latrines is aimed at pit latrine filling and nutrient recovery. For example, two shallow compost pit latrines designs, the Arborloo and Fossa Alterna have been developed [33, 34]. The importance of hygienic latrines has also been addressed. For example, a study by M Jenkins, et al. [35] noted that beyond the Millennium Development Goal’s definition of “improved” sanitation, hygienic safety and sustainability of the facilities was critical for their performance in low income urban areas of Dar es Salaam, Tanzania. In Kampala Uganda, it was found out that improved latrines failed to serve their purpose when misused or not properly cleaned [36, 37]. Other studies undertaken in urban slums of Kampala noted that understanding of the importance of using a clean toilet, the perceived disgust from using dirty toilets and user habits were essential in fostering users’ cleaning intention for shared toilets. Additionally, lack of cleanliness of latrines was linked to among other things, the lack of water or a lack of responsibility to buy the water to clean latrines, especially those that were shared [38–40]. Therefore, the availability of water and user intervention are important to assure latrine cleanliness.

### Pit latrine usage in urban areas of SSA

**Fig. 3:**
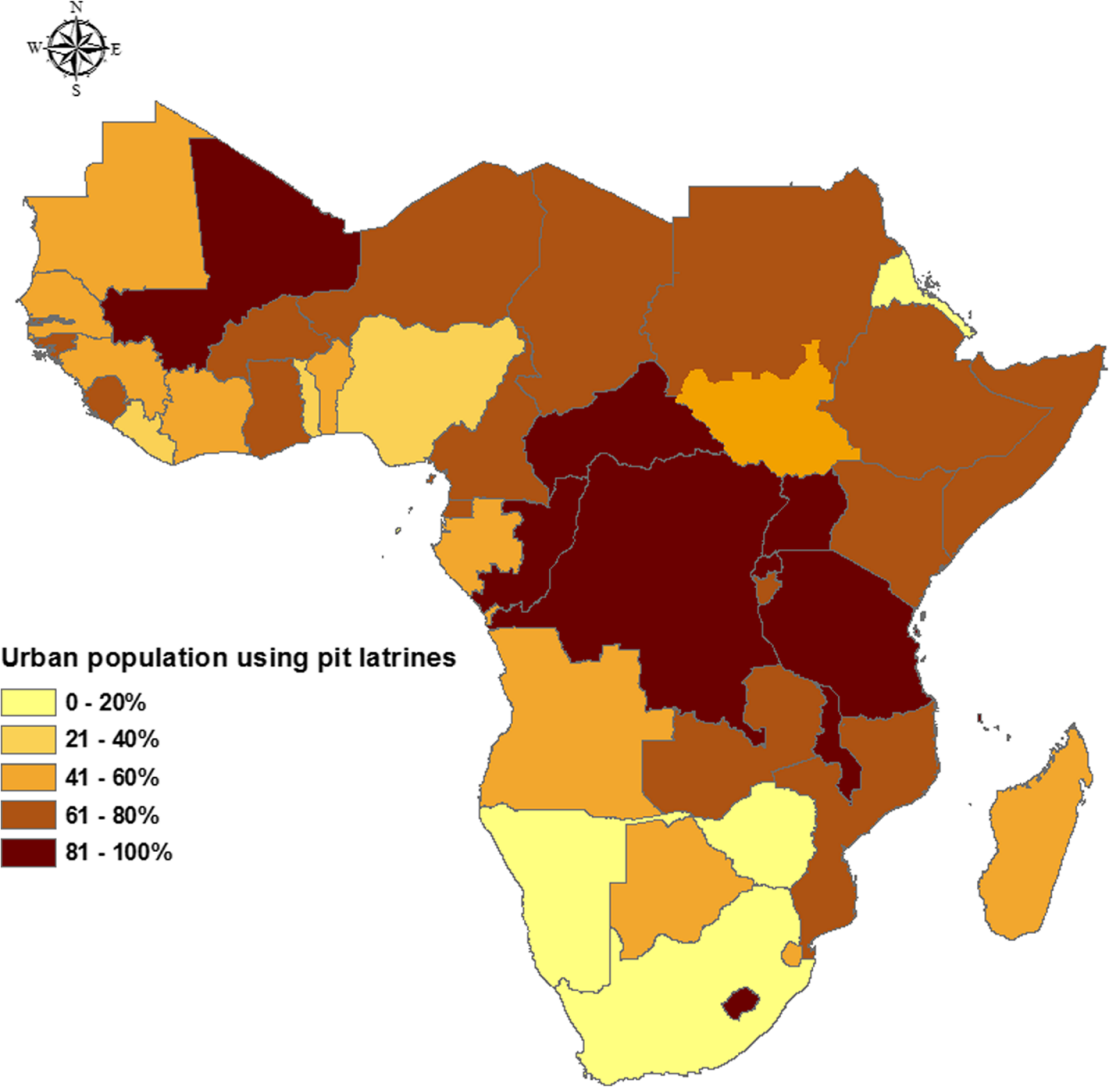
Percentage of SSA urban country populations using pit latrines [14]

Currently sanitation access for approximately 198 million (52.7 %) of the urban population in SSA is in form of a pit latrine (Additional file 1: Table S2). In 2007, pit latrine use in urban areas of SSA was at 65 %, representing about 162 million people [41]. While the percentage of pit latrine users has gone down (from 65–52.7 %) since 2007, the actual number of people using them has risen. An additional 36 million people have since adopted this sanitation technology since 2007. This number is expected to be higher as some of the percentages used during the calculations (Additional file) are from past years. The usage of pit latrine in SSA varies notably within the different countries (Fig. 3), and dramatically across the socio-economic spectrum, but is predominant among the low income earners [42].

**Fig. 4:**
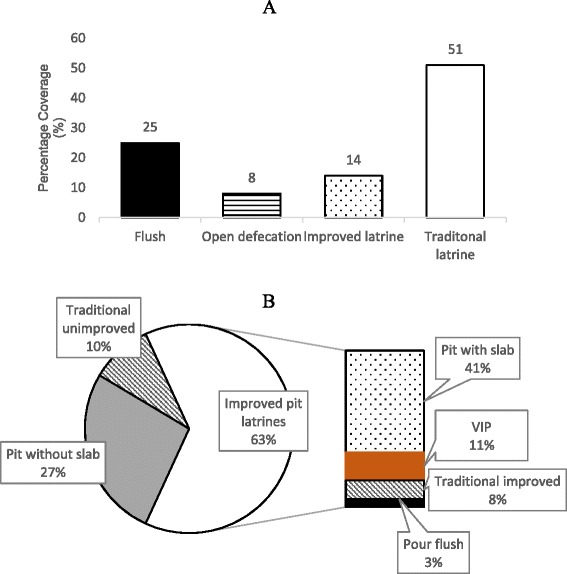
Percentage of pit latrine types in use in SSA. **a** usage in 2007 E Morella, et al. [42] and **b** usage in 2015 WHO and UNICEF [14]

The types of pit latrines being used within the urban areas of different SSA countries also vary. Presently, access to improved pit latrines is notably high. Overall usage of improved pit latrines stands at about 63 %, up from 14 % noted in 2007 (Fig. 4a and b). A number of countries have moved from the use of traditional pit latrines to more improved types (Additional file 1: Table S3). The commonest improved type is the simple pit latrine with a concrete slab. However, usage of VIP and pour flush latrines still remains low. In addition, there is still usage of pit latrines without slabs in urban SSA (Fig. 3b). The increase in access to improved pit latrines can be explained by the high awareness and action on sanitation from 2008 onwards [2].

### Sanitation policy and practice on pit latrine

One of the challenges of sanitation provision in the past was the little attention given to it and lack of clear policies to guide its provision. In the recent years, sanitation improvements have been at the forefront of most of the water and health projects [2, 23, 29]. There has been high political awareness within the international system, which has led to a number of strategies and policy reforms to address sanitation improvements. Different levels of service of pit latrines and other human excreta disposal facilities have been defined, based on the extent to which they provide improved sanitation and costs. VIP, and pit latrines with a slab are considered improved while pit latrines without slabs are considered unimproved [2]. However, while sanitation policies now exist in a number of countries in SSA, they state broadly the different sanitation technologies with no emphasis on minimum service levels of specific groups and technical details. For example, a review of policies from nine countries noted that only South Africa, Mozambique and Ghana had a VIP as their minimum sanitation standard. Additionally most policies do not allow for funding of sanitation technologies at household level [43, 44]. It has also been noted that sanitation service delivery is done via a multi-level process involving a number of actors [45] of which on site sanitation provision at household level is the responsibility of the owners. These often have limited knowledge of technical aspects on pit latrines [46]. In addition, the type of pit latrine adopted, is in most cases determined by socio- economic status of the owner. For example, where government involvement has been high, improvements in sanitation have been realised. One such case is Rwanda, where political will was successfully leveraged as all sanitation governance levels [45], and improved pit latrine coverage now stands at 82.2 % [14].

### Performance of pit latrines

There is a clear link between proper excreta disposal and improved health [20]. The appropriateness of pit latrines at providing improved sanitation thus lies in its ability to safely dispose human excreta in such a way that there is minimal or no contact with humans. Furthermore, the excreta should not be accessible to insects or animals and the facility should be free from odours [18, 47]. Research directly linking full pit latrines, their smell and insect nuisances to disease and health is limited. However, it has been reported that full and/or over flowing improved pit latrines do not meet the criteria for hygienic, safe and sustainable sanitation systems [35]. It is not only difficult to use full or overflowing pit latrines as the waste splashes on to the users but also the excreta poses a health risk since it is in closer contact with humans. Additionally, smell and insects nuisances of pit latrine use are the main cause of disturbance of people who come in contact with them. In the past, smell and insects significantly affected the user satisfaction, although the problem did not impact on pit latrine use [19, 48]. More recently, bad smell has been frequently mentioned as a reason for dissatisfaction with shared toilets [40, 49], discouraging their use and subsequent use of polyethylene bags [37]. Foul smell has also been noted as a barrier for acquiring and using latrines [50]. Smell and insects have been associated with the hygienic nature of the pit latrine. For example, in a survey by IK Tumwebaze and H-J Mosler [51], respondents considered clean latrines as those free from smell and insects. The subsequent sections detail pit latrine performance in terms of filling, smell and insect nuisances.

#### Pit latrine filling

Pit latrine filling is currently a problem associated with their performance. Notably the first faecal sludge management seminar was held in March 2011 in Durban, South Africa and brought to light issues related to pit latrine filling [52]. One of the concerns of pit latrine filling is that a number of the pit latrines within urban areas of SSA have reached their storage capacity. For example, VIPs built in Zimbabwe from 1980–2000 were reported to be full or nearly full [53]. A study by BF Bakare [54] reported that the number of pit latrines built across South Africa’s municipalities were full or over flowing. In Durban, South Africa alone, 35,000 pit latrines were emptied by 2011 [55]. In a study undertaken in informal settlements of Kampala, Uganda I Günther, et al. [56] noted that 35 % of the pit latrines had been abandoned because they had filled up while 15 % of the latrines were full and still in use. A study by E Appiah-Effah, et al. [57] undertaken in the Ashanti region of Ghana reported that 31 % of the latrines were found full and needed immediate de-sludging. M Jenkins, et al. [35] noted that 40 % of the latrines were full or nearly full in Dar es Salaam, Tanzania.

In the past, a pit latrine once full, was covered and a new one dug nearby. Double alternating pits were also proposed for use in peri-urban areas as they sanitize and reduce the volume of human excreta prior to emptying and disposal [26]. However, due to the high population density in most urban areas of SSA, digging new replacement pits and the use of alternate pits are not practical. Pit latrines can thus no longer serve as a stand-alone solution to human excreta management. A systems approach to sanitation is currently being adopted for urban settings to ensure their sustainability. In this case, the provision of access to improved sanitation is considered a multi-step process where a pit latrine is part of the chain, to be supported by the collection and transportation as well as treatment for safe end-use or disposal [58].

Attention is currently being focused on the time it takes for the pit to fill, since it is crucial for the management and sustainability of pit latrines. The actual filling times of pit latrines as noted in literature vary (Table 1). The available information indicates that pit latrines are mainly filling faster than expected. This has been attributed to the rate at which sludge accumulates within the pit. Most of the studies determining sludge accumulation have been based on number of users, filling time and the size of the pit. Proposed design accumulation rates range from 40–90 ℓ/capita/year [15, 18]. More recent field investigations undertaken in peri-urban South Africa by J Norris [59] found lower rates and thus proposed 25.5 ℓ/capita/year. In another study by DA Still [60] in South Africa, sludge accumulation rates were found to range between 10–120.5 ℓ/capita/year. Further studies by DA Still and K Foxon [61] noted filling rates of 1–264 ℓ/capita/year. Available data indicate variable pit latrine sludge accumulation/filling rates by region, even in a comparatively homogeneous environment (Table 2).

**Table 1:** Summary of studies on pit latrine filling time

Source	Country	Filling (Years)	Remarks
R Franceys, et al. [15]	Various	15–25	Design recommendations for household properties
J Pickford [17]	East Africa	Over 30	Reported at a house hold level
P Morgan [53]	Zimbabwe	Over 30	Household latrine
DA Still and K Foxon [107]	South Africa	20	Design recommendation
	South Africa	5–9	Empting time for most (85 %) pit latrines. Lower and higher filling rates were also noted
I Günther, et al. [56]	Uganda	5	Study in low income areas of Kampala, Uganda (Slums)
RN Kulabako, et al. [108]	Uganda	<1	Low laying areas of peri-urban settlements in Kampala
K Adubofour, et al. [109]	Ghana (slums in Kusami metroplis)	4.2	Average filling time
		>10	High income areas
		0.25	Low income areas
E Appiah-Effah, et al. [57]	Ghana (Ashanti region)	6–10	Low income area in Ashanti region

**Table 2:** Design accumulation rates and actual excreta filling rates

Design/Place	Filling rates litres/capita/annum (l/c/a)	Reference
Design accumulation rates		
EG Wagner and JN Lanoix [16] and R Franceys, et al. [15]	40	Wet pits where degradable anal cleansing material is used
	60	Wet pits where non degradable anal cleansing material is used
	60	Dry pits where degradable anal cleansing material is used
	90	Dry pits where non degradable anal cleansing material is used
Reported pit latrine filling rates		
EG Wagner and JN Lanoix [18] and R Franceys, et al. [15]	25 (ablution water used) 35 Wet pit	West Bengal, India
EG Wagner and JN Lanoix [18]	40 (solid cleansing material)	Philippines
PR Morgan, et al. [57]	20	Zimbabwe PR Morgan, et al. [94]
R Franceys, et al. [15]	42	USA
	47	Brazil
JN Bhagwan, et al. [110]	24.1 (mean)	Soshongove, South Africa
	69.4 (mean)	JN Bhagwan, et al. [110] Bester’s Camp, South Africa
	18.5 (mean)	Mbila, South Africa
	27.5 (implied)	Gabarone, Dares salaam
	29 (median)	Mbazwana, South Africa
	34 (median)	Inadi, South Africa
DA Still and K Foxon [53]	39 (median)	Limpopo, South Africa
	48 (median)	Mafunze, South Africa
	21 (median)	Ezimangweni, South Africa
	19 (mean)	eThekwine, South Africa

To explain the variation in sludge accumulation rates, studies have assessed different variables (Table 3) some of which are user related, like number of users, other material put in the pit and design related (type of pit latrine, lined or un lined), geophysical and climatic factors. Studies relating sludge accumulation rates to number of users have reported contrasting results. It is perceived that the filling rate increases with number of users. However, some field studies have reported a decrease in sludge accumulation rates with an increase in number of users [54, 61]. Additionally, BF Bakare [54] based on a linear model fit to the amalgamated data documented by DA Still and K Foxon [61], showed no significant correlation (Pearson correlation coefficient of 0.203) between sludge accumulation rate and number of users. However, it is important to note that this study was based on small increments from 5 to about 15 pit latrine users. The case could be different in urban settings where pit latrine sharing leads to higher number of users.

**Table 3:** Summary of studies assessing sludge accumulation rates, with different variables

Source	Country	Variable of interest	Study/experimental design	Remarks
DA Still and K Foxon [61]	South Africa	Number of users	Field monitoring and measurements	A decrease in per capita filling rate with an increase in number of users.
		Rubbish content	Sorting and analysis of pit content	Throwing rubbish in a pit almost doubled its filling rate
BF Bakare [54]	South Africa	Number of users	Analysis of amalgamated data documented by DA Still and K Foxon [61]	No correlation (Pearson correlation coefficient of 0.203) between sludge accumulation rate and number of users.
			Field monitoring and measurements	Sludge accumulation rates decreased with increasing numbers of users.
		Degradation	Laboratory experiments on pit latrine samples	50–70 % volume reduction in matter added to the VIP
		Addition of moisture	laboratory batch experiments on pit latrine samples	No evidence that an increase in moisture content of samples from VIP latrines reduced the sludge accumulation rate.
LC Todman, et al. [64]	Tanzania	Seasonal variation	Field monitoring and measurements	During wet periods, large temporary increases in the level (1 m magnitude) of pit content was observed
		Pit latrine Modelling	Modelling pit latrine filling based on model developed by C Brouckaert, et al. [63]	Water inflows and accumulation have an important effect on the filling rate
J Norris [59]	South Africa	Seasonal variation	Field monitoring and measurements	No effect of season variations on the sludge build up
EG Wagner and JN Lanoix [18]	Various	Degradation		A possible volume reduction of up to about 80 % after well-established degradation in wet pits
CA Buckley, et al. [62]	South Africa	Addition of moisture	Laboratory experiments on pit latrine samples	a significant increase on gas production rate was noted
		Increasing Alkalinity	Laboratory experiments on pit latrine samples	No statistically significant increases in the rate of gas production from the samples under anaerobic conditions.
		additives	Laboratory experiments on pit latrine samples	Inconclusive results
C Brouckaert, et al. [63]	South Africa	Pit latrine Modelling	Developing and testing a simple mass balance model	Adding non-degradable material to the pit significantly influenced its filling
K Foxon, et al. [71]	South Africa	additives	Laboratory experiments on pit latrine samples	No statistically significant effect on rate of mass loss
L Taljaard, et al. [69]	South Africa	Bio additives	Laboratory studies on pit latrine samples	Use of biological product is feasible
M Jere, et al. [70]	Zimbabwe	Spore forming bacteria	Pit latrine studies	Efficient in reducing pit content
FF Kassam [72]		Earthworm (Tiger worms)	Laboratory experiment setup	Reduction in human excreta
I Banks [74]	South Africa	Black soldier fly larvae	Laboratory studies on pit latrine samples	Potential in reduction of pit latrine content

Relating sludge accumulation to matter other than human excreta found that the degree of abuse to which the pit is subject affects the filling rate. Throwing rubbish in a pit almost doubled its filling rate in studies undertaken in South Africa [61, 62]. A simple mass balance model of pit latrine filling developed and tested by C Brouckaert, et al. [63] using data from VIPs in South Africa, predicted that adding non-degradable material to the pit significantly influenced its filling. A study by J Norris [59] noted no effect of seasonal variations on sludge accumulation in pit latrines in South Africa. However, in Tanzania, a large temporary increase in pit content was observed in the wet periods [64]. The ability of the model developed by C Brouckaert, et al. [63] to simulate data collected in south-central Tanzania and a sensitivity analysis of its parameters was tested by LC Todman, et al. [64]. The results indicated that water inflows and accumulation have an important effect on the filling rate. In Kampala (Uganda), a study relating the status of pit latrine structures to their performance noted that signs of rain or storm water entry, flooding and cleaning time were significant predictors of pit latrine filling [65]. This implied that water input into the pit significantly contributed to an increase in the level of pit content.

The rate of filling has also been attributed to the degradation processes occurring within the pit latrine over time. Matter starts to decompose as soon as it is deposited in the pit. Studies have depicted that the process of decomposition in pit latrines is largely anaerobic although aerobic degradation processes may occur [18, 62, 66]. During decomposition, the degradable fraction of faecal matter will break down into a more stable non-odorous product. Released gases flow into the atmosphere and mineral compounds are assimilated into the ground respectively. Through this action, the volume of matter added to the pit is substantially reduced [15, 48]. A possible mass - volume reduction of 50–75 % [54] or up to 80 % [18, 67] after well-established degradation has been reported. However, literature indicates that the uncontrolled environment within the pit may not be efficient for decomposition under either process which results in slow/incomplete breakdown of organic matter [68].

In order to quantify the role of decomposition and stabilization on mass loss within pit latrines, laboratory batch experiments have been undertaken. Addition of moisture to samples of pit content in laboratory experiments had a significant increase on gas production rate [62]. It was thus concluded that increasing moisture content of VIP contents has the potential to increase the rate of stabilisation of buried organic material in the pit. However, in a study by BF Bakare [54] no evidence was found to show that an increase in moisture content of samples from VIP latrines reduced the sludge accumulation rate. The study proposed that compaction could play an important role on the rate at which pits fill up. The effect of increasing alkalinity (addition of Sodium bicarbonate), thereby the pH buffering capacity of pit latrine samples was assessed by CA Buckley, et al. [62]. The increase in the rate of gas production from the samples observed under anaerobic conditions was not statistically significant. It was thus concluded that alkalinity was not a limiting factor in anaerobic digestion of pit latrine contents.

Studies on inoculation with additives, which are reportedly a mixture of various microorganisms, some blended with enzymes said to enhance degradation of pit content have also been undertaken. Relatedly, L Taljaard, et al. [69] reported a feasibility in the use of biological products for the degradation of organic matter. However, the study was inconclusive and recommended field trials to daily monitor contents of newly dug pits. A biological study into the claimed mode of action of the products, to determine the amount and type of microorganisms and enzymes present was also proposed. Earlier, M Jere, et al. [70] studied the effects of spore forming non-pathogenic bacteria in reducing sludge volume in pit latrines and concluded that the bio-organic breakdown compound proved to be efficient in reducing the pit contents. However, CA Buckley, et al. [62] obtained no correlation in decrease of faecal matter between the used additives and the rate of change in pit matter content. The results were considered inconclusive due to the difficulty in obtaining representative measurements of any condition and lack of test control sites. Furthermore, K Foxon, et al. [71] reported no statistically significant effect on the rate of mass loss from the sludge samples under either aerobic or anaerobic conditions by nine additives. It was concluded that commercial pit latrine additives did not accelerate the rate of decomposition of pit latrine contents. Subsequently, DA Still and K Foxon [61] concluded that sufficient evidence was lacking to prove that pit latrine additives could cause differences in pit latrine sludge build-up.

Earth worms have also been investigated for their potential to reduce pit latrine contents with successful results [72]. Currently, they are the basis of the tiger toilet, a worm- based sanitation technology aimed at speeding up the decomposition of human waste [73]. Black soldier fly larvae (BSFL), *Hermetia illucens* has also shown potential in reducing pit latrine sludge. Research by I Banks [74] found the characteristics of faecal sludge from different pit latrines in South Africa to be within the range for BSFL development. Key factors that affected the faecal mass reduction were moisture and larvae density. However, further research is required on the applicability of these organisms in pit latrines.

#### Pit latrine odours and insect nuisance

The extent of the smell and insect nuisance found in literature has mainly been listed by intensities based on a pre- determined scale (Table 4). Only two studies listed the odour descriptions associated with particular pit latrine smell intensity (Table 5). Of the listed intensities, the strong, unpleasant, repugnant, foul, malodorous smell and any presence of flies are of importance in pit latrine performance.

**Table 4:** Pit latrine odour intensity and description

Source	Location	Smell description (%)	Insect nuisance (%)
A Cotton, et al. [19]	Ghana and Mozambique (Simple pit latrines and VIPs respectively)	No smell (54 and 40)	None/tens (91 and 90)
		Slight smell (9 and 6)	Hundreds (8 and 3)
		Strong smell (37 and 51)	Thousands (1 and 7)
J Kwiringira, et al. [37]	Kampala’s slums	Strong repugnant smell	
JV Garn, et al. [111]	Kenyan schools	Strong smell (25.6)	Many flies (10)
A Nakagiri, et al. [65]	Kampala’s slums	No smell, (2)	No flies (3)
		Slight smell (35)	Few flies (80)
		Moderate smell (22)	Many flies (17)
		Strong smell (39)	
		Very strong (1)	
K Afful, et al. [112]	Kusumi, Ghana	Extremely annoying (69 no)	
		Very annoying (55 no)	
		Annoying (30 no)	
		Some annoyance (18 no)	
		Definitely not annoying (1 no)

**Table 5:** Pit latrine odour intensity and description

Source	Site	Pit latrine type	Odour intensity	odour description
J Lin, et al. [76]	Durban	VP dry pit	Weak	Sewage, phenol-like
			strong	Rotten egg, sewage, rancid
		VP wet pit	Medium	More of sewage than faecal, rotten egg
			Strong	Rotten egg, sewage, rancid
	Nairobi	VP	strong:	cheese, manure, horse, farmyard
			Strong	cheese, manure, ammonia, urine
	Kampala	VP 1	weak	farmyard, ammonia slightly urine, geosmin (earthy, moisture)
			strong	rancid, rotten onion, phenylacetic acid-like
		VP 2	medium	farmyard, ambrinol (earthy, moisture), rancid
			strong	rancid, phenolic, rotten vegetable
CJ-Fo Chappuis, et al. [113]	Nairobi		Weak	barnyard
	Durban	VIP	Weak	Animal, faecal

Information on the actual composition of the malodorous gases in pit latrine is limited. Methane, carbon dioxide, nitrogen, ammonia and hydrogen sulphide have for long been noted as the smell causing substances in pit latrines [18, 75]. However, a study by J Lin, et al. [76] using gas chromatography - mass spectrometry and olfactive analyses found many more odorants. Of the 198 volatile constituents detected [77], isobutyric, butyric, isovaleric, 2methyl butyric, valeric, hexanoic and phenylacetic acids were responsible for the rancid, cheesy odour/smell in pit latrines. The manure, farmyard, horse-like characteristics of latrine odour were attributed to the combined effects of phenol, p-cresol, indole, skatole, and some carboxylic acids. Dimethyl sulphide, dimethyl disulphide, dimethyl trisulphide, methyl mercaptan, and hydrogen sulphide were contributed to the sewage, rotten egg, and rotten vegetable odours. The sewage malodourous smell in pit latrines has been attributed to anaerobic degradation while the rancid odour was noted to be representative of latrines dominated with fresh faeces [76]. Fermenting urine resulting from enzymatic cleavage of urea by ureases has been noted to be representative of the smell found in public pit latrines [78, 79].

Unlike smell, studies characterising insects in pit latrines have been undertaken. Adult and larvae of *Chrysomya putoria*, *Chrysomya marginalis*, *Musca spp*, *Lucilia cuprina*, *Sarcophaga spp* have been reported [80–82]. S Irish, et al. [83] identified members of *Psychodidae*, *Culicidae*, *Calliphoridae*, *Syrphidae*, *Stratiomyidae*, *Sarcophagidae* families from pit latrines in central Tanzania. Some types of mosquitoes especially *Culex quinquefasciatus* and species of Anopheles are known to breed in wet pits [84, 85].

Studies have linked the presence of odours and insects in pit latrines to the type and size of the superstructure, and cleanliness. S Irish, et al. [83] noted that the superstructure minimises the fly nuisance in pit latrines. Absence of a roof for example significantly associated with presence of flies. In addition more flies have been found in latrines with temporary structures. In Kampala Uganda, latrines that were not regularly cleaned were associated with bad smells [40] and caused disgust among the users [39]. Another study noted that pit latrine cleanliness, stance length, superstructure material and single household use were predictors of smell. Fly presence was predicted by the superstructure material and status, plus the terrain where the pit latrines were located [65]. Entomological studies on pit latrines in Botswana and Tanzania [80] linked the insect nuisance to the smell. The studies showed that insects in pit latrines were attracted by the odours as many flies and mosquitoes were caught trying to enter the vent pipe which indicated they were drawn to the smell source.

Addressing the odour and insect nuisance of pit latrines has involved simple recommendations like the concrete slab that is easily cleaned and ensuring that the pit remains dark during use, which is achieved partly by the use of hole/ seat covers [18]. The use of inorganic and organic chemicals as larvicides and disinfectants like sodium fluosilicate, borax, paradichlorobenzene (PDB), orthodichlorobenzene (ODB), aldrin, BHC and DDT has been documented [17, 18, 86]. Muscabac, a *Bacillus thuringiensis* preparation containing exotoxin, was tested and showed reasonably good control of flies in latrines in a tropical environment [87]. Household surveys have also reported addition of oil, kerosene, ash, soil, and disinfectants to control odour and insects [88–90]. Laboratory and field experiments on the use of expanded and shredded waste polystyrene beads to eliminate mosquitoes in pit latrines have been very successful [91]. Traps placed over the squatting plate hole have also been developed and experimented with success at controlling insects in pit latrines [82]. Pyriproxyfen, an insect juvenile hormone, and local soap have been found to reduce flies in pit latrines [92].

Improvements in the design of the pit latrine have also been done to minimise the smell and fly nuisance. Incorporation of a vertical vent pipe with a fly trap and the natural effect of the sun and wind are the principle mechanisms for the functioning of a VIP latrine. The design makes use of circulation of air from outside the latrine, through the superstructure into the pit, then up and out of the vent pipe thereby exhausting any odours emanating from the faecal material in the pit via the vent pipe [75, 93]. The superstructure is kept dark to prevent flies from going into the latrine. The top of VIP vent pipe is fitted with a wire mesh fly-screen that prevents any flies inside the pit from escaping via the vent pipe where they die and fall back into the pit.

Experiments on the performance of VIP latrines in Zimbabwe showed that they were effective in smell and fly control compared to identical unvented pit latrines. However, the ventilation system was not as effective at mosquito control [94]. This was because while both flies and mosquitoes were drawn to odour sources in pit latrines, [80] the latter have a positive phototropism and fly only towards light [18]. Contrary to the studies in Zimbabwe, field investigations undertaken by A Cotton and D Saywell [48] in Ghana and Mozambique that were based on a user’s perceptions recorded a higher degree of odour nuisance with the use of VIPs. In a recent study undertaken on pit latrines in Kampala Uganda, VIPs did not provide superior performance (smell, flies) to the simple pit latrines. Additionally, logistic regression showed that VIPs are not likely to smell less nor have fewer flies than simple pit latrines [65]. This was attributed to the VIPs not meeting minimum design standards, and overcrowding in the slums that could have impeded ventilation within the VIPs to achieve odourless conditions.

In order to understand the mechanisms inducing ventilation in the VIP design, field studies were undertaken in Botswana and Zimbabwe. PR Morgan, et al. [94] found that the action of the wind blowing across the top of the vent pipe induced ventilation. The effect of solar heating the vent was only negligible [75]. Additionally, satisfactory odour control in VIP latrines was achieved with a ventilation rate of 10 m^3^/h and 6 superstructure air volume changes / h (ACH). A more recent study by JW Dumpert [95] on VIP latrines in the upper west region of Ghana found out that mechanisms driving ventilation were air buoyancy forces resulting in a stack effect at times in which ambient temperatures are less than temperatures inside the pit of the latrine; and suction wind passing over the mouth of the vent pipe and when possible wind passing into the superstructure. The study further noted that, majority of the latrines (73 %) achieved ventilation flow rates greater than 10 m^3^/h. However, the flow rates were not adequate enough to achieve the 6 ACH as to maintain odourless conditions. The larger volume of the pit latrine superstructures in this study compared to those found in Botswana and Zimbabwe was noted to contribute to the low ACH. Additionally the vent pipe sizes were found to be inadequate, while most structures were constructed with openings and entrances facing away from the wind direction.

Other design improvements to the simple pit latrine that have been noted in literature to improve the odour and smell nuisance include the SanPlat pit latrine which consists of a thin circular dome shaped slab of the pit with no reinforcement and has a removable lid cast in the squat hole to ensure it fits tightly. Contrary to the VIP latrine where air is encouraged to flow through the structure, the SanPlat prevents air in and out flows of the pit. The opening into the pit is always kept tightly closed when not in use. Thus most odours remain within the pit and are assumed to be absorbed by the pit walls [28]. A pit latrine modification with a specially made bowl incorporated in the ordinary concert slab uses a water seal to control odour and insects. About 1–2 L of water is usually poured by hand into the bowl to flush faecal matter into the pit [15].

### Knowledge gaps and way forward

The use of pit latrines in urban areas of SSA is high and constantly rising. However their performance in terms of filling, smell and insect nuisance is not satisfactory. Available literature shows that contributions have been made to address shortfalls related to pit latrine use. However further research within this area is needed. Knowledge gaps that can be identified from this review include:

1. Sludge accumulation within pit latrines is a function of a number of variables. A clear understanding of sludge accumulation within pit latrines is essential. The use of sludge accumulation rates based on users to determine pit sizes is not sufficient. Determining the exact number of users in highly populated areas is difficult. Incidentally, pit latrines also receive additional material other than human excreta and anal cleansing material. Collecting information on the actual pit sludge accumulation rates in different settings, taking into account other material applied in pits during their use is important. This will in turn guide prediction of sustainability and aid in better pit latrine designs.
2. Research into processes taking place in pit latrines is still new and limited. While sludge accumulation has been related to moisture, alkalinity and additive inoculum, the actual contents and factors that account for the decomposition process in pit latrines cannot be conclusively stated. It has been indicated that the decomposition process is variable and the environment of the pit is uncontrolled and is affected by the design, usage and geophysical and climatic factors. Additionally, the decomposition process is responsible for the smell and insect nuisances of pit latrines. There is need to understand the content and environment within the different pit latrine types. Furthermore, an understudying of organic matter decomposition, degradation pathways and fundamental factors controlling their occurrence and their relation to filling, smell and insects is essential.
3. Microorganism inoculums, earthworms and black soldier fly larvae have been used in degradation of organic matter with varying levels of success. However, the success in their application is strongly linked to the need for having the right organism biomass and optimization of the essential environmental factors as the environment should not deviate tremendously from the optimal growth range of the strain biomass in the inoculum [96, 97]. In the case of pit latrines, additives have been developed without a clear understanding of the content and environmental characteristics in the pit, yet they could affect the physio-chemical and biological processes of the additives used. Additionally, the composition of pit latrine additives and their optimal operation conditions are not known.
4. The smell and insect nuisances need be clearly quantified. Currently odour meters have been invented that can be used to give different levels of smell. A clear understanding of the composition of pit latrine smell is essential so as to help find solutions to its reduction. Such techniques have been used in the perfume industry with success. However, as there maybe limitation on adaptation of the smelling techniques from the perfume industry in the study of pit latrines, obtaining clear representative gases for smell in pit latrines could help in research for their reduction.
5. The smell and insect nuisance in pit latrines are closely associated. The association arises because flies are attracted by the smell from the pits [18], while volatile compounds from pit latrines function as pheromones to attract gravid mosquitoes to suitable breeding sites [98–101]. Skatole (3-methylindole) Indole 4-metylindole have been cited as the most active pheromones attracting mosquitoes [102–105]. By eliminating the active pheromones compounds in the gases emitted from pit latrines, the insect nuisance can be mitigated.
6. Determination of the appropriate superstructure sizes and construction materials for pit latrines is also essential, as these have been found to affect smell and insects within pit latrines. This will also help in developing standards for pit latrine designs.
7. Beyond technology development and process management, proper construction and maintenance of pit latrines is essential. The importance of hygienic sanitation facilities has been demonstrated, and this is largely dependent on the users. Additionally, currently urban sanitation polices lack specification of minimum technology option and service standards. Besides enforcement of sanitation policies is often lacking [106]. As household owners are unaware of alternative or better functioning pit latrine designs the quality of pit latrines constructed has been greatly compromised. To improve the situation, there is need to develop, disseminate and enforce pit latrine technology specifications and service standards for different target groups and to sensitise on the need for hygienic latrines.

## Conclusions

The pit latrine is a sanitation technology that has been in use for a long time and the design has evolved over time. The technology is used by majority of the people in SSA, while its use in the urban areas is currently on the rise. The current trend of usage shows adaptation of more improved designs. From this review, it can be deduced that the performance in pit latrines in terms of filling, smell and insects within urban areas is an issue that needs further investigation.

Future advances in pit latrine technology should focus on scientifically guided approaches to enhanced and sustainable sanitation. A precursor of understanding the content, environment, decomposition process, smell/ odour and insect composition is essential in predicting and favourably altering the conditions within the pit through technological novelty or process management. In addition, development, dissemination and enforcement of minimum pit latrine design standards for target groups is important while the importance of hygienic latrines should also be emphasized.

## Additional file

Additional file 1: Table S1. Summary of success and failure attributes of different sanitation technologies used in Sub-Saharan Africa. **Table S2.** Summary data on pit latrine use in urban areas of Sub-Saharan Africa. **Table S3.** Comparison of 2015 and 2007 pit latrine coverage figures. (DOC 157 kb)
