# Supplementary material for: Are pit latrines in urban areas of Sub-Saharan Africa performing? A review of usage, filling, insects and odour nuisances
Source: BMC Public Health. 2016 Feb 4;16:120. doi: 10.1186/s12889-016-2772-z (PMC4743102; doi:10.1186/s12889-016-2772-z)
Supplement: Additional file 1: Table S1. — Summary of success and failure attributes of different sanitation technologies used in Sub-Saharan Africa. Table S2. Summary data on pit latrine use in urban areas of Sub-Saharan Africa. Table S3. Comparison of 2015 and 2007 pit latrine coverage figures. (DOC 157 kb) [file 12889_2016_2772_MOESM1_ESM.doc]

**SUPPLEMENTARY MATERIAL**

Table S 1 Summary of success and failure attributes of different sanitation technologies used in Sub-Saharan Africa

| **Sanitation technology** | **Attributes of success** | **Attributed of failure** |
| --- | --- | --- |
| Septic tank | - Offers a high standard of hygiene - Requires little mechanical maintenance - Permanent, emptied and reused | - High cost of installation - Shortage of space - Blockages - Water shortage |
| Aqua privies | - Requires less land - No pipes, less liable to blockages. | - Bad smells/odours, - Requires large volumes of water |
| Biogas latrines | - Provides biogas for energy, - Slurry produced is a good plant nutrient | - High installation costs - High technical skill to operate and maintain - Cultural phobia regarding slurry management |
| Composting/ dehydrating toilets | - Pits are re-usable, conserves space - Excreta contained, sanitized and can be recycled in agriculture | - Lack of spare parts for maintenance - High technical skill to operate and maintain. - High cost of repairs |
| Pit latrines | - Low cost of construction - Simple technology - Little water needed for operation - Easy to operate and maintain - Easily upgraded | - Filling up and thus need for space and money to build new ones - Bad smells/odours - Harbors insects and vermin |

Table S 2 Summary data on pit latrine use in urban areas of Sub-Saharan Africa

| **Country** | **Data Source** | **Report year** | **Flush Toilet**  **(%)** | **Pit Latrine usage (%)** | **Pit latrine usage by type (%)** | | | | | |  |
| --- | --- | --- | --- | --- | --- | --- | --- | --- | --- | --- | --- |
|  |  |  |  |  | **Pour flush** | **VIP** | **Pit latrine with slab (covered pit)** | **Traditional latrine** | **Pit latrine without slab (open pit)** | **Traditional latrines considered improved** | **2015 Population**  **(Thousands)** |
| Burundi | DHS-MIS | 2012 | 34.3 | 64.6 | 0.0 | 0.0 | 32.9 | 25.6 | 6.0 | 14.6 | 1,305 |
| Comoros | EIM | 2004 | 14.9 | 83.7 | 0.0 | 0.0 | 35.2 | 19.2 | 29.2 | 0.0 | 219 |
| Eritrea | DHS | 2002 | 41.8 | 18.8 | 0.0 | 3.2 | 0.0 | 15.6 | 0.0 | 7.8 | 1,564 |
| Ethiopia | DHS | 2011 | 7.2 | 72.2 | 0.0 | 3.3 | 31.7 | 0.0 | 37.1 | 18.6 | 17,873 |
| Kenya | MIS | 2010 | 29.4 | 65.4 | 0.0 | 26.2 | 0.0 | 39.2 | 0.0 | 19.2 | 11,985 |
| Madagascar | MIS | 2011 | 16.7 | 49.3 | 0.0 | 0.3 | 3.9 | 0.0 | 39.0 | 19.5 | 8,512 |
| Mauritius | CEN | 2011 | 99.6 | 0.7 | 0.1 | 0.0 | 0.0 | 0.3 | 0.0 | 0.2 | 525 |
| Rwanda | DHS | 2010 | 5.6 | 92.9 | 0.0 | 3.1 | 79.1 | 0.0 | 10.7 |  | 2,530 |
| Somalia | MICS | 2005 | 22.6 | 69.8 | 41.3 | 1.6 | 12.5 | 0.0 | 14.4 |  | 4,410 |
| South Sudan | CEN | 2008 | 1.0 | 41.5 | 0.0 | 0.0 | 0.0 | 41.5 | 0.0 | 20.6 | 2,289 |
| Sudan | MICS | 2010 | 21.5 | 66.9 | 0.0 | 10.6 | 28.3 | 0.0 | 28.0 |  | 13,405 |
| Uganda | DHS | 2011 | 11.6 | 86.2 | 0.0 | 17.2 | 39.1 | 22.6 | 5.5 | 11.3 | 6,930 |
| United Republic of Tanzania | LSMS | 2011 | 27.7 | 88.7 | 20.7 | 6.4 | 35.3 | 26.3 | 0.0 |  | 14,953 |
| Benin | DHS | 2012 | 12.4 | 54.8 | 0.0 | 4.4 | 35.8 | 0.0 | 13.0 |  | 5,169 |
| Burkina Faso | DHS | 2010 | 7.0 | 79.6 | 0.0 | 1.8 | 72.8 | 0.0 | 5.0 |  | 5,352 |
| Côte d'Ivoire | DHS-MIS | 2012 | 44.6 | 47.5 | 0.0 | 0.0 | 31.6 | 0.0 | 15.9 |  | 11,536 |
| Cameroon | DHS | 2011 | 20.7 | 77.6 | 0.0 | 2.0 | 60.8 | 0.0 | 14.5 |  | 12720 |
| Cape Verde | CEN | 2010 | 74.2 | 22.0 | 21.6 | 0.0 | 0.0 | 0.4 | 0.0 | 0.2 | 333 |
| Equatorial Guinea | MICS | 2000 | 30.3 | 69.7 | 32.7 | 0.0 | 29.0 | 0.4 | 0.0 | 0.2 | 321 |
| Gambia | MICS | 2010 | 33.7 | 65.8 | 0.0 | 5.9 | 51.5 | 0.0 | 8.4 |  | 1,171 |
| Ghana | DHS | 2011 | 29.1 | 60.9 | 0.0 | 40.0 | 10.7 | 0.0 | 9.7 |  | 14,702 |
| Guinea | DHS | 2012 | 45.7 | 53.7 | 0.0 | 3.0 | 37.4 | 0.0 | 13.1 |  | 4,619 |
| Guinea-Bissau | MICS | 2010 | 22.7 | 74.6 | 4.4 | 0.0 | 0.0 | 62.3 | 0.0 | 7.9 | 833 |
| Liberia | DHS | 2011 | 40.9 | 38.4 | 0.0 | 4.6 | 8.3 | 0.0 | 15.1 |  | 2,239 |
| Mali | MICS | 2010 | 13.2 | 84.3 | 0.0 | 0.8 | 59.5 | 0.0 | 24.0 |  | 6,100 |
| Mauritania | MICS | 2007 | 35.2 | 51.5 | 0.0 | 11.5 | 21.6 | 0.0 | 18.4 |  | 1,739 |
| Niger | LSMS | 2011 | 8.0 | 76.3 | 0.0 | 0.0 | 29.0 | 27.4 | 19.9 | 13.7 | 3,637 |
| Nigeria | MICS | 2011 | 50.5 | 37.3 | 0.0 | 2.2 | 25.9 | 0.0 | 9.0 |  | 95,564 |
| Sao Tome and Principe | DHS | 2009 | 40.6 | 6.6 | 0.0 | 6.6 | 0.0 | 0.0 | 0.0 |  | 132 |
| Senegal | DHS | 2013 | 43.2 | 54.1 | 0.4 | 11.7 | 34.0 | 8.0 | 0.0 | 4.0 | 6,554 |
| Sierra Leone | OSM | 2011 | 15.7 | 77.6 | 0.0 | 4.1 | 45.1 | 0.0 | 27.9 |  | 2,576 |
| Togo | MICS | 2010 | 41.4 | 38.8 | 0.0 | 0.7 | 30.9 | 0.0 | 6.2 |  | 2,866 |
| Central African Republic | MICS | 2010 | 1.4 | 91.6 | 0.0 | 1.4 | 9.7 | 0.0 | 18.3 |  | 1926 |
| Chad | MICS | 2010 | 18.5 | 63.1 | 0.0 | 1.0 | 33.3 | 0.0 | 24.7 |  | 3,019 |
| Congo | DHS | 2012 | 15.1 | 83.5 | 0.0 | 2.0 | 42.2 | 0.0 | 37.0 | 13.5 | 3053 |
| Democratic Republic of the Congo | MICS | 2010 | 12.6 | 83.1 | 0.6 | 0.7 | 0.0 | 21.4 | 60.4 | 11.1 | 25,996 |
| Gabon | DHS | 2012 | 38.0 | 59.1 | 0.0 | 6.2 | 26.7 | 0.0 | 22.3 |  | 1,530 |
| Angola | MICS | 2011 | 42.3 | 45.2 | 0.0 | 2.2 | 36.2 | 0.0 | 6.0 |  | 14,193 |
| Botswana | BAIS | 2008 | 41.0 | 56.0 | 0.0 | 0.0 | 0.0 | 56.0 | 0.0 | 33.9 | 1,319 |
| Lesotho | CMS | 2012 | 11.1 | 84.0 | 0.0 | 42.9 | 24.0 | 0.0 | 17.1 |  | 646 |
| Malawi | DHS | 2012 | 13.7 | 82.3 | 0.0 | 7.2 | 24.1 | 0.0 | 51.0 |  | 2,822 |
| Mozambique | DHS | 2011 | 18.6 | 77.1 | 9.6 | 22.5 | 15.9 | 0.0 | 29.0 |  | 8,746 |
| Namibia | CEN | 2011 | 68.7 | 8.5 | 0.0 | 3.6 | 2.2 | 0.0 | 1.4 |  | 973 |
| South Africa | IES | 92.3 | 5.8 | 0.0 | 1.8 | 0.0 | 3.3 | 0.0 | 1.8 | 2.1 | 34,101 |
| Swaziland | MICS | 46.4 | 52.0 | 0.0 | 7.8 | 40.1 | 0.0 | 4.1 | 47.9 |  | 272 |
| Zambia | CEN | 31.9 | 67.8 | 0.0 | 2.6 | 0.0 | 65.2 | 0.0 | 2.6 | 49.0 | 6,352 |
| Zimbabwe | DHS | 88.2 | 9.8 | 0.0 | 3.1 | 3.1 | 0.0 | 1.9 | 6.2 |  | 6,106 |

Notes;

**Data source** DHS: Demographic and Health Survey, CEN: Census, MICS: Multiple Indicator Cluster Survey, MICS: Multiple Indicator Cluster Survey, AGVSAN: Analyse Globale de la Vulnérabilité, de la Sécurité Alimentaire et de la Nutrition, LSMS: National survey on household living conditions and agriculture, OSM Opportunities for Sanitation Marketing in Sierra Leone, BAIS Botswana Aids Impact Survey

Table S 3 Comparison of 2015 and 2007 pit latrine coverage figures

| **Country** | **Sanitation access 2015** | | | |  | **Sanitation access 2007** | | | |
| --- | --- | --- | --- | --- | --- | --- | --- | --- | --- |
|  | **Flush Toilet** | **VIP/ pit latrine with slab/ pour flush latrines/ SANPLAT** | **Traditional latrine** | **Pit latrine without slab (open pit)** | **2015 Population**  **(Thousands)** | **Flush Toilet** | **VIP/ Ventilated toilet/ SANPLAT** | **Traditional pit latrine** | **Population 2007**  **(Thousands)** |
| Benin | 41.4 | 31.6 | 0.0 | 6.2 | 5169 | 6.0 | 29.0 | 26.0 | 3683 |
| Burkina Faso | 7.0 | 74.6 | 0.0 | 5.0 | 5352 | 8.0 | 70.0 | 14.0 | 3297 |
| Cameroon | 20.7 | 62.8 | 0.0 | 14.5 | 12720 | 16.0 | 41.0 | 41.0 | 9499 |
| Central African Republic | 1.4 | 11.1 | 0.0 | 18.3 | 1926 | 2.0 | 6.0 | 86.0 | 1574 |
| Chad | 18.5 | 34.3 | 0.0 | 24.7 | 3019 | 7.0 | 12.0 | 64.0 | 2317 |
| Comoros | 14.9 | 35.2 | 19.2 | 29.2 | 219 | 8.0 | 35.0 | 56.0 | 176 |
| Congo | 15.1 | 44.2 | 0.0 | 37.0 | 3053 | 10.0 | 25.0 | 62.0 | 2326 |
| Côte d'Ivoire | 44.6 | 31.6 | 0.0 | 15.9 | 11536 | 30.0 | 23.0 | 44.0 | 8663 |
| Democratic Republic of the Congo | 12.6 | 1.3 | 21.4 | 60.4 | 25996 | 4.0 | 26.0 | 60.0 | 12518 |
| Ethiopia | 7.2 | 35.0 | 0.0 | 37.1 | 17873 | 8.0 | 4.0 | 77.0 | 12949 |
| Gabon | 38.0 | 32.9 | 0.0 | 22.3 | 1530 | 32.0 | 27.0 | 40.0 | 1224 |
| Ghana | 29.1 | 50.7 | 0.0 | 9.7 | 14702 | 23.0 | 39.0 | 27.0 | 11067 |
| Guinea | 45.7 | 40.4 | 0.0 | 13.1 | 4619 | 8.0 | 3.0 | 87.0 | 3382 |
| Kenya | 29.4 | 26.2 | 39.2 | 0.0 | 11985 | 39.0 | 11.0 | 44.0 | 8464 |
| Lesotho | 11.1 | 66.9 | 0.0 | 17.1 | 646 | 8.0 | 38.0 | 45.0 | 483 |
| Madagascar | 16.7 | 4.2 | 0.0 | 39.0 | 8512 | 7.0 | 67.0 | 6.0 | 5820 |
| Malawi | 13.7 | 31.3 | 0.0 | 51.0 | 2822 | 18.0 | 2.0 | 74.0 | 2089 |
| Mali | 13.2 | 60.3 | 0.0 | 24.0 | 6100 | 15.0 | 21.0 | 62.0 | 4117 |
| Mauritania | 32.2 | 33.1 | 0.0 | 18.4 | 1739 | 4.0 | 9.0 | 66.0 | 1353 |
| Mozambique | 18.6 | 48.0 | 0.0 | 29.0 | 8746 | 8.0 | 5.0 | 68.0 | 6732 |
| Namibia | 68.7 | 5.8 | 0.0 | 1.4 | 973 | 79.0 | 2.0 | 5.0 | 752 |
| Niger | 8.0 | 29.0 | 27.4 | 19.9 | 3637 | 5.0 | 55.0 | 21.0 | 2420 |
| Nigeria | 50.5 | 28.1 | 0.0 | 9.0 | 95564 | 28.0 | 5.0 | 58.0 | 69281 |
| Rwanda | 5.6 | 82.2 | 0.0 | 10.7 | 2530 | 6.0 | 48.0 | 43.0 | 1789 |
| Senegal | 43.2 | 46.1 | 8.0 | 0.0 | 6554 | 65.0 | 9.0 | 22.0 | 4944 |
| South Africa | 92.3 | 1.8 | 3.3 | 0.0 | 34101 | 80.0 | 0.0 | 9.0 | 29852 |
| South Sudan | 1.0 | 0.0 | 41.5 | 0.0 | 2289 | 14.0 | 0.0 | 60.0 | 12518 |
| Sudan | 21.5 | 38.9 | 0.0 | 28.0 | 13405 |  |  |  |  |
| Togo | 41.4 | 31.6 | 0.0 | 6.2 | 2866 | 0.0 | 33.0 | 35.0 | 2107 |
| Uganda | 11.6 | 56.3 | 1.9 | 5.5 | 6930 | 11.0 | 9.0 | 78.0 | 4298 |
| United Republic of Tanzania | 27.7 | 62.4 | 26.3 | 0.0 | 14953 | 10.0 | 12.0 | 75.0 | 10280 |
| Zambia | 31.9 | 2.6 | 65.2 | 0.0 | 6352 | 47.0 | 2.0 | 47.0 | 4535 |
| Zimbabwe | 88.2 | 6.2 | 0.0 | 1.9 | 6106 | 95.0 | 2.0 | 2.0 | 4681 |
